# Female Radiological Metatarsal Morphometry and Diameter Suitability of Intramedullary Implants Used for Midfoot Charcot

**DOI:** 10.1155/aort/7497747

**Published:** 2025-10-13

**Authors:** Kaissar Yammine, Youssef Jamaleddine, Anthony ElAlam, Joseph Mouawad, Ahmad Haj Hussein, Taha Khaled, George Sayegh, Chahine Assi

**Affiliations:** ^1^ Department of Orthopedic Surgery, Lebanese American University Medical Center-Rizk Hospital, School of Medicine, Lebanese American University, Beirut, Lebanon, lau.edu.lb; ^2^ Department of Health Sciences, University of Texas, Austin, Texas, USA, uta.edu

**Keywords:** diabetic charcot, intramedullary implant, metatarsal bones, morphometry

## Abstract

**Objective:**

Radiological biometrics of the metatarsal have been reported in few studies but mostly with no relevance to clinical settings. The smaller female metatarsal diameter could be an impediment for intramedullary implants. This study investigates female radiological metatarsal morphometry for diameter suitability of intramedullary implants used for midfoot Charcot fusion.

**Methods:**

Standing foot roentgenograms of 143 female patients were collected for radiological measurements. Cortical thickness at the metatarsal neck level was calculated by subtracting inner width from outer width values. After hypothetical reaming, inner width, outer width, and cortical thickness mean values were considered for suitability estimation. Implant suitability was deemed acceptable when (a) inner width values more than the cut‐off value of 6 mm and/or (b) cortical thickness more than the cut‐off value of 2 mm (1 mm on each cortex side).

**Results:**

The results were (a) inner width: M5 had a higher mean value when compared to M2, M3, and M4 (*p* < 0.00001) with no difference between central metatarsals and (b) outer width: M2 showed a significantly higher outer width than M3 and M4 (*p* < 0.00001) with no difference with M5. Based on the cut‐off values, the simulated reaming width values to accept implant diameter were found unsuitable for M2, M3, and M4 and the estimated cortical thickness values were found unsuitable for M3, M4, and M5.

**Conclusion:**

Our study indicates for the first time a mismatch between the inner width of the lateral metatarsals at the neck level with the commercially available Charcot‐specific IM implants for female patients.

## 1. Introduction

Radiological biometrics of the metatarsal have been reported in few studies but mostly with no relevance to clinical settings [1, 2]. Some ‘‘forensic’’ articles reported sex‐based morphometric metatarsal length values and found significant differences between men and women [3–5]. Other metatarsal bone indexes were found to be more reliable for sex identification. For example, the metatarsal width was more accurate than the metatarsal length in determining the gender [6, 7].

From a clinical perspective, morphometric studies of metatarsals could help assess the severity of disorders, planning surgeries and better choosing implants when needed. For instance, morphometric data of metatarsals could have a high clinical relevance when treating the midfoot deformities of conditions like diabetic Charcot arthropathy. With a prevalence ranging between 0.8% and 7.5% [8], diabetic Charcot arthropathy has a weighted 5‐year mortality frequency of 24.5% and a weighted amputation frequency of 15% [9]. The midfoot is the most affected site (60%) probably due to the substantial forces during the transfer of weight from the hindfoot to the forefoot [10]. When the deformity is severe or the conservative management fails, surgical reconstruction of the midfoot using intramedullary (IM) implants such as bolts, beams, and screws is needed. However, the rate of failure was found to be high. Out of 279 feet, Wukich et al. reported that 24.4% of cases required reoperation, 19.4% developed nonunion, and hardware complications occurred in 26.2% with breakage in 11.5% and migration in 14.7% of cases [11]. Interestingly, when compared to non‐Charcot specific implants, Charcot‐specific implants were found to yield significantly worse outcomes in terms of reoperation, nonunion, and hardware migration [11].

Implant migration in midfoot Charcot has been related to many variables such as the match of thread diameter with shaft implant diameter, thread surface area of purchase, and thread length [11]. An uninvestigated variable, the mismatch between the implant diameter and the intramedullary canal diameter mainly in female patients, could be a possible cause of implant migration. In the lateral metatarsals, the available commercial implants are sometimes inserted with some difficulty with reports of cortical fractures due to their higher diameter compared to the diameter at the level of the neck of the metatarsal [11]. Cortical breach or fracture is a well‐known complication of IM nail insertion of long bones, where it is usually due to suboptimal entry point or under‐reaming [12, 13].

A lack of precise estimation of the radiological morphometry of the metatarsals and its unknown correlation with medullary implants used to treat midfoot Charcot could be one factor among others that could lead to surgical failure [11]. This gap has been identified by our team earlier [14]. Therefore, the aim of the study was to investigate the female radiological morphometry of the metatarsals and to estimate their suitability to the available intramedullary implants used to treat midfoot Charcot.

## 2. Materials and Methods

### 2.1. Study Design

This is a retrospective radiological observational study conducted at the Lebanese American University Medical Center. All patients who were surgically treated for hallux valgus were included in the study. Electronic charts and electronic x‐rays between 2019 and 2022 were reviewed and inclusion/exclusion criteria were applied. Inclusion criteria were as follows: (a) only female patients with hallux valgus deformity, no history of foot fracture or other deformity or previous surgery, and standardized radiograms in a standing position. Exclusion criteria include male patients, unclear, or nonstandardized radiograms. Approval of the study was given prior to its conduction by the Institution Review Board (LAUMCRH.KY1.6/Apr/2023).

### 2.2. Parameters and Outcomes

**Figure 1:**
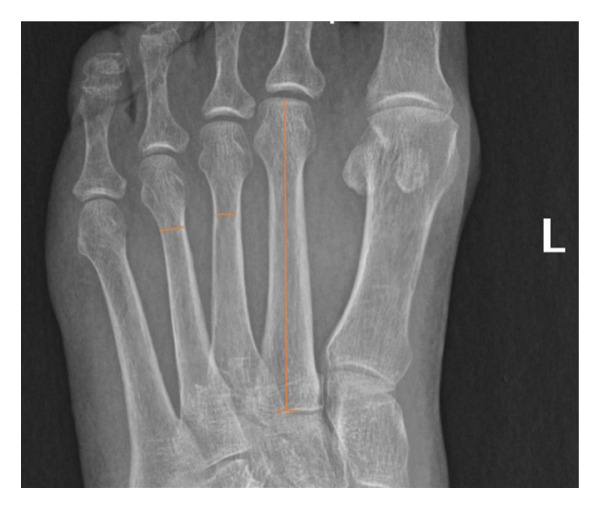
Measurements of length (M2), inner (M3), and outer (M4) metatarsal widths of a left foot.

Basic biometric parameters on anteroposterior weight‐bearing views were first calculated: length, inner width, outer width, and cortical thickness. Measurements were done using the tools of the Picture Archiving and Communication System (PACS, v12.2.1.2, USA). The length is defined as the distance projected from the uppermost of the metatarsal head to the metatarsal base (shown on M2 of Figure 1). The inner width is the distance between the inner cortices at the level of the distal shaft, as shown on M3 of Figure 1. The outer width is the distance between the outer cortices at the level of the distal shaft as shown on M3 of Figure 1. A junior resident performing the measurement in the presence of a senior resident who was checking the accuracy of the measurement process.

The primary outcome was set as the simulated reaming width value, calculated through a hypothetical mathematical simulation to account for the effects of reaming on the inner width and cortical thickness of the metatarsals. It was set that reaming a metatarsal would remove 1 mm from each cortex side for an easy implant insertion, while it would add 2 mm to the radiological inner width value. Though it is admitted that reaming is typically 1.5 mm larger than the planned implant for long bones [13], a reaming of 2 mm could be more appropriate since IM implants for metatarsals are advanced by rotational movements rather than tapping.

The hypothetical reaming values of the inner width and cortical thickness would define the estimated implant suitability. The mathematical simulation model method was as follows: simulated reaming width = inner width + cortical thickness lost postreaming. The mean values of the cortical thickness for each metatarsal were calculated by subtracting the inner width mean values from the outer width mean values.

Since the least implant diameter available commercially is of 5 mm, it was defined that the cut‐off value of the estimated width to be higher than 6 mm for implant suitability. In addition, to avoid cortical breach, the cut‐off value of the cortical thickness was set to be higher than 2 mm (meaning more than 1 mm on each side) for implant suitability.

### 2.3. Statistical Analysis

Means ± standard deviation (SD) and values were reported for each parameter. ANOVA was conducted to look for mean differences between metatarsals. Post hoc Tukey test was used to look for potential type 1 errors for pairwise comparisons. Lower quartile values were used to further analyze the subset of patients (25% of the sample) with the lowest values. *p* values less than 0.05 were considered as significant. StatsDirect (Cambridge, UK, version 3.3.6) software was used for all statistical analyses.

## 3. Results

The sample consisted of 143 female patients (240 feet) with a mean age of 52 ± 14.8 years. Side distribution was as follows: 119 right and 121 left.

### 3.1. Basic Biometric Values

Table 1 summarizes all biometric parameter values.

**Table 1:** Parameters’ and outcomes’ values (in mm).

Variables	Statistic	1^st^ metatarsal	2^nd^ metatarsal	3^rd^ metatarsal	4^th^ metatarsal	5^th^ metatarsal
Length	Mean ± SD	55.5 ± 3.8	64.6 ± 5	62.5 ± 5.1	63.8 ± 4.5	63.6 ± 3.8

Inner width	Mean ± SD	9.2 ± 1.2	3.5 ± 0.9	3.4 ± 0.7	3.6 ± 0.7	4.6 ± 0.8
	Lower quartile	≤ 8.3	≤ 3.03	≤ 2.9	≤ 3	≤ 4.07

Outer width	Mean ± SD	12.8 ± 1.4	7.6 ± 0.8	6.6 ± 0.7	6.6 ± 0.7	8 ± 1.8
	Lower quartile	≤ 11.9	≤ 7.1	≤ 6.1	≤ 6.1	≤ 7.4

Cortex thickness	Mean ± SD	3.5 ± 1.9	4.1 ± 1.2	3.2 ± 1.05	3 ± 1.02	3.3 ± 2.04
	Lower quartile	≤ 2.3	≤ 3.3	≤ 2.5	≤ 2.3	≤ 2.5

Inner width after hypothetical reaming	Mean	7.2	5.5	5.4	5.6	6.6
	Lower quartile	6.7	≤ 5	≤ 4.9	≤ 5	≤ 6.07

Cortex thickness after hypothetical reaming	Mean	1.5	2.1	1.2	1	1.4
	Lower quartile	≤ 0.6	≤ 1.3	≤ 0.5	≤ 0.3	≤ 0.5

#### 3.1.1. Length

M1 was significantly shorter than all other metatarsals (*p* < 0.00001). M2 length was significantly higher than M3 (*p* = 0.00002). Though the mean of M2 length was higher than M4, it was not statistically significant. M3 was significantly shorter than M4 (*p* = 0.02), but it was not significantly shorter than M5. No statistical difference was found between lengths of M4 and M5 (*p* = 0.4).

#### 3.1.2. Inner Width

M1 presented with a significantly higher inner width than all other metatarsals (*p* < 0.00001). M5 had a higher mean value when compared to M2, M3, and M4 (*p* < 0.00001). No statistically significant differences were found between the mean values of inner width of M2, M3, and M4.

#### 3.1.3. Outer Width

M1 presented with a significantly higher outer width than all other metatarsals (*p* < 0.00001). M2 showed a significantly higher outer width than M3 and M4 (*p* < 0.00001) and no significantly difference with M5. No statistically significant differences were found between the mean values of outer width of M3 and M4. M5 had a significantly higher mean value when compared to M3 and M4 (*p* < 0.00001).

#### 3.1.4. Cortical Thickness

The reported values represent the sum of both cortices (Table 1) that means that the thickness of each cortex has the half value.

### 3.2. Calculating Inner Width and Cortical Thickness Values After Hypothetical Reaming

In the hypothesis that reaming would remove 1 mm on each cortical side, the simulated reaming width values would be higher by 2 mm since the cortical thickness (the subtraction of inner width from outer width values) would lose 2 mm following reaming (Table 1).

### 3.3. Simulated Reaming Width

Hypothetical reaming generated inner width values of less than 6 mm for M2, M3, and M4. The cortical thickness of M3, M4, and M5 had values of less than 2 mm, mostly close to 1 mm, and only M2 had a cortical thickness value higher than 2 mm (2.1 mm). Lower quartile values showed that 25% of the sample had an inner width value of less than 5 mm. Further, 25% of the sample had a cortical thickness value much less than 2 mm (Table 1).

## 4. Discussion

To our knowledge, this is the first study to look for the suitability of the IM metatarsal canal with regard to the available commercial implants used in Charcot deformity. Our study showed, for female lateral metatarsals, a mismatch in at least 25% of patients, of width values between those following simulated reaming and the available implants in the market. Therefore, a higher risk of cortical fracture and implant migration could be anticipated. Such results could incite manufacturers to take into consideration the gender difference in width values for midfoot arthrodesis.

This study highlights three morphometric and anatomical parameters that could be relevant to clinical practice. The inner width, level of measurement, and reaming simulation are factors that could impact the performance of arthrodesis when treating Charcot midfoot deformity using IM implants. In addition, in order to enhance clinical relevance and ensure the broad applicability of IM implants for all patients, female feet were chosen for analysis where relevant parameters are known to have smaller values compared to men [2–7].

The studied three parameters were not reported in other studies. First and besides length measurements, only the outer width at mid‐diaphyseal level, away from the narrowest IM level, was recorded. For instance, Patil et al. and Abdel Moneim et al. found the widest metatarsal to be M1, followed by M2, M5, M4, and M3, respectively [2, 3]. Dogan et al. showed that outer width values of M2 and M5 were equal [1]. Calculation of the inner along with the outer width values for cortical thickness evaluation would give more insight to the suitability of the diameters of available implants. Second, the level of metatarsal neck between the metatarsal diaphysis and the distal metaphysis, area of the smallest diameter, is more relevant for the diameter of IM implants. Third, a simulation of the impact of reaming, as a necessary step to widen the IM canal [11], on the residual width was conducted in pursue of better accuracy for implant suitability.

Our analysis revealed that the final estimated inner width after reaming was less than the cut‐off value of 6 mm, ranging between 5.4 and 5.6 mm, for M2, M3, and M4. The residual cortical thickness after reaming showed values well below the cut‐off value of 2 mm for M3, M4, and M5. To add, a further analysis using the lower quartile values demonstrated that 25% of the patients had inner width values less than 5 mm for M2, M3, and M4 and 25% had cortical thickness slightly above 1 mm for M3, M4, and M5.

Considering practical implications, it is crucial to note that the smallest available bolts (such as Wright Medical Salvation, Milwaukee, USA) or beams (like Redemption, Tennessee, USA) have a diameter of 5 mm (Table 2). In light of our results, this poses a challenge for surgeons to properly accommodate the IM implants in the lateral female metatarsals. These findings highlight the potential risk for cortical fracture when inserting the available implants in female patients in general and most likely in men having values less than the male average value.

**Table 2:** Commercial implants with the smallest diameter size.

Implant name	Diameter size (mm)
Wright medical salvation bolt, Milwaukee, US	5
DePuy Synthes bolt, Indiana, USA	6.5
Stryker IM nail, Michigan, USA	8
Lavender medical bolt, Hertfordshire, UK	5.5
Redemption nail, Tennessee, USA	7.5
Redemption beams, Tennessee, USA	5

### 4.1. Implication for Practice

This study would recommend measuring the width of the lateral metatarsal necks preoperatively as an important planning step, mainly in women. In instances where a diameter mismatch is identified, two viable alternatives are available: (a) inserting the implants through an opening on the proximal aspect of the lateral metatarsals, where the metatarsal basis provides sufficient space to accommodate the implant diameter, or (b) opting for cannulated IM screws with a diameter of less than 5 mm, providing a more suitable alternative in cases where the available commercial implants are not compatible. The second option implicates a critical low implant diameter with the known consequence of higher odds of implant breakage [15–18]. These recommendations might be extrapolated to any patient, male or female, with a critical preoperative estimated diameter suitability.

### 4.2. Implications for Research

Our study highlights the importance of conducting large‐sampled observational studies to validate our findings. It also highlights the need for prospective comparative trials between men and women to detect the fraction of men having relevant morphometric values below the cut‐off values. Since anatomic variations could be ethnicity‐based [19], comparative studies between different ethnic populations could improve the morphometric human database. In addition, the results of this study should incite researchers to investigate new implant design with more adapted diameter for the lateral metatarsals without compromising the stiffness of the construct that could induce implant failure.

### 4.3. Limitations

No comparison with male values was conducted in our study. As some studies have shown [6, 7], metatarsal width values are higher in males compared to females; however, males with lesser width values could benefit from the results of this study. Another limitation could be the hypothetical statement that reaming would remove 2 mm (1 mm of each cortical side) from the metatarsal inner width. The value was based on the fact that reaming long bones for IM nailing is usually 1.5–2 mm higher than the planned diameter of the implant [20]. However, manual reaming might impact width/cortex values differently from powered reaming. Future studies with different reaming types in cadavers or intraoperatively could give more accurate values on thickness loss values following IM reaming. Our sample included women with hallux valgus surgery. It may be argued that results related to the first metatarsal could be impacted by the valgus deformity; however, the main aim of this study is focused on the lateral metatarsals where width suitability could be critical for IM implants. Furthermore, no studies analyzing morphometric changes of lateral metatarsals in the presence of hallux valgus could be found. Charcot that require surgical stabilization is frequently associated with dislocation at the tarso‐metatarsal level; therefore, measurement accuracy of metatarsal morphometry could be affected by such deformity which impedes plantigrade assessment of antero‐posterior standing x‐rays. Age has been incriminated in metatarsal morphometry changes, but results were inconclusive [21–23]. While the cross‐sectional properties of diaphyseal bones seem to be mainly affected by physical activity before skeletal maturity [7], our sample mean age was similar to the age, approximately 60 years [20], of diagnosis of Charcot foot.

CT scan imagery might have generated more accurate measurements compared to roentgenogram imaging. Further studies using CT scan for metatarsal morphometry could be of interest to validate our findings.

## 5. Conclusion

Our study demonstrated a mismatch between the width of the lateral metatarsals at the neck level with the commercially available Charcot‐specific IM implants for female patients. The residual values of inner width and thickness of the cortices after an eventual reaming could be critical for a proper insertion of the implant through the MTP joint. Novel implant designs are needed in the future to accommodate for the female and for some male metatarsal width.

## Ethics Statement

The Ethical Institutional Committees granted the approval prior to the conduction of the study.

## Conflicts of Interest

The authors declare no conflicts of interest.

## Author Contributions

All authors contributed to the article and approved the submitted version. Kaissar Yammine: conception, design of the study, data analysis, and writing first and final draft. Anthony ElAlam: data collection and writing final draft.

Youssef Jamaleddine: data collection and writing final draft.

Joseph Mouawad: writing first draft and data collection.

Ahmad Haj Hussein: data collection and writing first draft.

Taha Khaled: data collection and writing first draft.

Chahine Assi: supervision and writing final draft.

## Funding

No funding was received for this manuscript.

## Data Availability

Data could be accessed upon request from the corresponding author.
